# Retraction: Standardised Mindfulness-Based Interventions in Healthcare: An Overview of Systematic Reviews and Meta-Analyses of RCTs

**DOI:** 10.1371/journal.pone.0215608

**Published:** 2019-04-12

**Authors:** The PLOS ONE Editors

In this article [1], the authors presented a systematic overview of 23 systematic reviews and pooled results from the 8 (of the 23) reviews that reported meta-analyzed standardised mean differences (SMD) of five outcomes. After the article was published, it came to light that the handling Academic Editor shared an affiliation with three of the authors. Due to this potential competing interest, the *PLOS ONE* Editors had the article reassessed by another member of the journal’s Editorial Board. During this reassessment concerns were raised about pooling of meta-analytic results in the meta-analysis, i.e. the authors considered each meta-analysis as an individual study in the analysis, rather than pooling results of individual RCTs as per community standards for this type of study. Since some randomized clinical trials (RCTs) were included in more than one meta-analysis, this pooling resulted in double counting, incorrect effect estimates in Fig 1A, 1B and 1E, and incorrect confidence intervals.

The authors noted that they had set a double counting maximum *a priori* in the meta-analyses, and excluded reviews that reported duplicate data until the predefined limit (10% per outcome) was reached, as explained in the Methods section (S3 Table identified the double counted RCTs per pooled outcome).

On re-evaluation of the data post-publication, the authors found two additional errors. In the supplementary table the two RCTs from the Veehof et al. 2011 meta-analysis were inadvertently not listed and in Fig 1 the authors had inadvertently included in pooled estimates of anxiety the meta-analyses of Cramer et al. 2012 and Galante et al. 2012, which led to double-counting beyond the predefined maximum.

The study’s pooled estimates and confidence intervals for the outcomes stress and quality of life did not include any double counted results. However, this issue impacted the other results reported in the article, and the consulted Academic Editor advised that a full re-analysis would be needed, in which individual RCTs rather than meta-analyses are pooled, in order to correctly address the study’s aims.

In addition, we note here that the following information was missing from the article’s Competing Interests statement: Herbert Benson and Gregory Fricchione hold or have held positions at the Benson-Henry Institute for Mind Body Medicine at Massachusetts General Hospital, which is paid by patients and their insurers for running the SMART-3RP and related relaxation/mindfulness clinical programs, markets related products such as books, DVDs, CDs and the like, and holds a patent pending (PCT/US2012/049539 filed August 3, 2012) entitled “Quantitative Genomics of the Relaxation Response.”

In light of the methodological issue and concerns about the validity of the study’s results, the *PLOS ONE* Editors retract this article. We regret that these issues were not fully addressed prior to the article’s publication.

RAG, PC, JJVB, HB, GLF, MGMH did not agree with retraction.
